# Safety and immunogenicity of the *Na*-APR-1 hookworm vaccine in infection-naïve adults

**DOI:** 10.1016/j.vaccine.2022.09.017

**Published:** 2022-10-06

**Authors:** David J. Diemert, Maria Zumer, Doreen Campbell, Shannon Grahek, Guangzhao Li, Jin Peng, Maria Elena Bottazzi, Peter Hotez, Jeffrey Bethony

**Affiliations:** a Department of Medicine, School of Medicine and Health Sciences, The George Washington University, Washington DC, USA; b Department of Microbiology, Immunology and Tropical Medicine, School of Medicine and Health Sciences, The George Washington University, Washington DC, USA; c Departments of Pediatrics, Division of Pediatric Tropical Medicine, and Molecular Virology and Microbiology, Texas Children's Hospital Center for Vaccine Development, National School of Tropical Medicine, Baylor College of Medicine, Houston, TX, USA

**Keywords:** Hookworm, *Necator americanus*, *Na*-APR-1, Immunization, Glucopyranosyl lipid A, Immune responses, Safety

## Abstract

**Background:**

The *Necator americanus* hemoglobinase, aspartic protease-1 (*Na*-APR-1), facilitates the ability of adult hookworms to parasitize the intestine of their human hosts. A recombinant version of APR-1 protected laboratory animals against hookworm infection by inducing neutralizing antibodies that block the protein’s enzymatic activity and thereby impair blood feeding. A catalytically inactive version of the wild-type hemoglobinase (*Na*-APR-1(M74)) was expressed by infiltrating *Nicotiana benthamiana* tobacco plants with an *Agrobacterium tumefaciens* strain engineered to express the vaccine antigen, which was adjuvanted with aluminum hydroxide adjuvant (Alhydrogel).

**Methods:**

An open-label dose-escalation Phase 1 clinical trial was conducted in 40 healthy, hookworm-naïve adult volunteers in the United States. Participants received 30 or 100 µg of recombinant *Na*-APR-1(M74) with Alhydrogel or with Alhydrogel co-administered with one of two doses (2.5 or 5.0 µg) of an aqueous formulation of Glucopyranosyl Lipid A (GLA-AF). Intramuscular injections of study vaccine were administered on days 0, 56, and 112.

**Results:**

*Na*-APR-1(M74)/Alhydrogel was well-tolerated; the most frequent adverse events were mild or moderate injection site tenderness and pain, and mild or moderate nausea and headache. No serious adverse events or adverse events of special interest related to vaccination were observed. Significantly higher levels of antigen-specific IgG antibodies were induced in those who received 100 µg *Na*-APR-1(M74) than those who received 30 µg of antigen. Adding GLA-AF to *Na*-APR-1(M74)/Alhydrogel resulted in higher levels of IgG against *Na*-APR-1(M74) in both the 30 and 100 µg *Na*-APR-1(M74) groups in comparison to the non-GLA formulations at the same antigen dose.

**Conclusions:**

Vaccination of hookworm-naïve adults with recombinant *Na*-APR-1(M74) was well-tolerated, safe, and induced significant IgG responses against the vaccine antigen *Na*-APR-1(M74). Given these favorable results, clinical trials of this product were initiated in hookworm-endemic areas of Gabon and Brazil.

## 1 Introduction

Between 170 and 400 million people worldwide are infected with one of the two principal species of hookworm that infect humans, primarily in resource-limited areas of the tropics [1], [2]. *Necator americanus* is the most prevalent human hookworm species [3]. Chronic infection with *N. americanus*, which can last for years (even decades), may result in considerable morbidity due to the intestinal bleeding that is the main complication associated with this helminth [4]. High intensity infections have a greater likelihood of resulting in iron-deficiency anemia, which in turn is associated with impaired physical and cognitive development in children and adverse pregnancy outcomes in women of childbearing age [5]. The estimated number of disability-adjusted life years lost annually due to such sequelae of hookworm disease is>4 million [6].

Interventions to limit hookworm-related morbidity include regular mass drug administration of mebendazole or albendazole, both of which are benzimidazole anthelminthics [3]. However, this approach is unlikely to lead to elimination of hookworm for several reasons, not least of which is because of the inevitable re-infection that rapidly follows treatment due to ongoing exposure to the larval stage of the parasite in regions that lack adequate sanitation and treatment of human waste. Additionally, the development of drug resistance to the benzimidazole class of anthelminthics is a real threat, since nematode resistance to these drugs has been documented in veterinary medicine after extensive use in livestock [6]. An effective hookworm vaccine that would protect against moderate- and heavy-intensity infections with *N. americanus* could significantly reduce the morbidity attributed to hookworm [7], [8]. Modeling the epidemiologic and economic impacts of an efficacious hookworm vaccine indicates that it would be a cost-effective addition to the disease control toolbox [6].

After initial infection through the skin, hookworm larvae migrate through host tissues to the small intestine, where they mature into adult worms attached to the mucosa. Hookworms subsist in the intestine by consuming host blood and subsequently cleaving the hemoglobin that is released from lysed erythrocytes [9]. Adult *N. americanus* hookworms utilize a cascade of proteases to break hemoglobin down into small peptides that the helminth uses to meet its energy needs [9], [10], [11], [12], [13], [14]. The Aspartic Protease-1 enzyme of *N. americanus* (*Na*-APR-1) is responsible for initiating this proteolytic pathway. When used as a vaccine antigen, it is hypothesized that *Na*-APR-1 will induce antibodies that neutralize the catalytic activity of native *Na*-APR-1, interrupting the blood feeding of *N. americanus* and thereby either killing the worms or impairing egg production, both of which would also contribute to the interruption of transmission.

When *Na*-APR-1 is expressed as a recombinant protein for use as a vaccine antigen, two aspartic acid residues are substituted with alanines to produce the enzymatically inactive hemoglobinase, *Na*-APR-1_mut_, also known as *Na*-APR-1(M74). Canines vaccinated with recombinant *Na*-APR-1_mut_ were protected against development of anemia following heterologous challenge with *Ancylostoma caninum* [14]. More specifically, vaccinated dogs generated antibodies against *Na*-APR-1_mut_ that blocked the enzymatic activity of wild-type *Na*-APR-1 as visualized in the intestine of expulsed worms. Based on these encouraging preclinical results, the catalytically inactive version of *Na*-APR-1 was manufactured for clinical trials by infiltration of *Agrobacterium tumefaciens* strain GV3101 that was genetically modified to express *Na*-APR-1(M74) in *Nicotiana benthamiana* tobacco plants [15]. As reported herein, a first-in-humans Phase 1 trial of this vaccine antigen, formulated on the Alhydrogel aluminum hydroxide adjuvant, was conducted in the United States (US) in healthy adult volunteers without previous exposure to hookworm. Recombinant *Na*-APR-1(M74) adjuvanted with Alhydrogel was evaluated with or without the addition of one of two doses (2.5 or 5 µg) of an aqueous formulation of Glucopyranosyl Lipid A (GLA-AF), a Toll-like receptor (TLR)-4 agonist.

## 2 Materials and methods

### 2.1 Study vaccines

Catalytically inactive recombinant *Na*-APR-1(M74) was expressed, purified, and adsorbed to Alhydrogel (Biosector, Denmark) [15]. Vials of *Na*-APR-1(M74)/Alhydrogel contained recombinant protein (0.1 mg/mL) and Alhydrogel (0.8 mg/mL) in a 1.35 mL solution consisting of 10 mM imidazole, 0.3 % Empigen BB and 150 mM sodium chloride (pH 7.4 ± 0.1). During the trial described herein, immunogenicity studies of drug product stored at 2 to 8 °C were performed every 6 months to assess vaccine potency as part of ongoing stability studies.

GLA-AF was manufactured and supplied by the Infectious Diseases Research Institute (Seattle, WA) in multi-dose vials containing a 50 µg/mL aqueous solution of GLA without preservative. The *Na*-APR-1 (M74)/Alhydrogel plus GLA-AF formulations were prepared in a biosafety cabinet in the site investigational pharmacy by adding an appropriate volume of GLA-AF solution to a vial of *Na*-APR-1 (M74)/Alhydrogel®, or vice versa, within 24 h of vaccination.

### 2.2 *Phase 1 trial of* Na*-APR-1(M74)*

An open-label Phase 1 clinical trial of the *Na*-APR-1 Hookworm Vaccine was conducted in Washington, DC from 2013 to 2015. The primary objective was to evaluate the vaccine’s safety and reactogenicity in healthy adults aged 18 to 50 years, inclusive. Antibody responses to vaccination were assessed as secondary objectives. Exclusion criteria included the presence of clinically significant systemic disease; pregnancy or breast feeding; laboratory confirmation of chronic infection with hepatitis B, hepatitis C, or HIV viruses; receipt of corticosteroids or other immunosuppressive drugs; receipt of an inactivated vaccine within the 2 weeks prior to the first study vaccination, or a live vaccine within the prior 4 weeks; and, history of previous infection with hookworm or residence for>6 months in a hookworm-endemic area.

Forty volunteers were progressively enrolled into 2 cohorts. In cohort 1, volunteers were assigned to receive either 30 µg *Na*-APR-1(M74)/Alhydrogel (n = 5), 30 µg *Na*-APR-1(M74)/Alhydrogel co-administered with 2.5 µg GLA-AF (n = 5) or 30 µg *Na*-APR-1(M74)/Alhydrogel co-administered with 5.0 µg GLA-AF (n = 10). In cohort 2, participants received either 100 µg *Na*-APR-1(M74)/Alhydrogel (n = 5), 100 µg *Na*-APR-1(M74)/Alhydrogel co-administered with 2.5 µg GLA-AF (n = 5) or 100 µg *Na*-APR-1(M74)/Alhydrogel co-administered with 5.0 µg GLA-AF (n = 10).

Enrollment into the cohorts was staggered such that safety and reactogenicity data for the 8 days following the first vaccinations of cohort 1 were reviewed by the Safety Monitoring Committee (SMC) prior to initiating cohort 2 vaccinations. Additionally, enrollment within each cohort was staggered so that vaccine preparations containing 0, 2.5, and 5 µg GLA-AF were tested consecutively: those administered *Na*-APR-1(M74)/Alhydrogel combined with 2.5 µg GLA-AF were enrolled not<3 days following enrollment of the last volunteer to be vaccinated with the formulation containing no GLA-AF, while participants administered *Na*-APR-1(M74)/Alhydrogel plus 5 µg GLA-AF were enrolled not earlier than 7 days after the final volunteer received the 2.5 µg GLA-AF formulation.

Vaccinations were administered on study days 0, 56, and 112 by intramuscular injection in the deltoid muscle. Study product was administered in an open-label fashion given the dose-escalation design of the trial.

### 2.3 Ethics statement

The study was approved by the institutional review board (IRB) of the George Washington University and the trial was conducted after submission of an investigational new drug application (number BB-15290) to the US Food and Drug Administration. The trial was registered at Clinicaltrials.gov (NCT01717950). Informed consent was obtained in writing from all volunteers before any study activities or procedures.

### 2.4 Assessment of safety and tolerability

Study participants were observed for at least 1 h after vaccination to assess immediate reactogenicity. They were subsequently evaluated at 3, 7, 14, and 28 days after each vaccination for injection site and systemic reactogenicity, and then at regular intervals until 12 months after the third injection. At each study visit, the presence of injection site pain was queried, and injection sites were examined for swelling/induration, erythema, and tenderness. The systemic symptoms that were solicited included headache, fever, myalgia, arthralgia, rash, urticaria, nausea, and vomiting. Adverse event severity was defined as mild (easily tolerated), moderate (interfered with activities of daily living), or severe (prevented activities of daily living); causality in relation to study product was determined based on investigator judgement. Injection site swelling and erythema were assessed as mild (>25 to ≤ 50 mm in diameter), moderate (>50 to ≤ 100 mm), or severe (>100 mm); whereas oral temperature was graded as mild (≥38.0 °C to < 38.5 °C), moderate (≥38.5 °C to < 39.0 °C), or severe (≥39.0 °C). Serum alanine aminotransferase, creatinine and complete blood counts were done on the day of each vaccination as well as approximately 14 days later. Abnormal safety laboratory test results were assessed as mild, moderate, or severe according to standardized toxicity tables.

Active surveillance for the following adverse events of special interest was conducted throughout this clinical trial due to the use of the novel GLA-AF adjuvant, as requested by the US FDA: autoimmune musculoskeletal disorders (e.g., systemic lupus erythematosus, Sjögren’s syndrome, rheumatoid arthritis), neuroinflammatory disorders (e.g., multiple sclerosis, optic neuritis, Guillain-Barré syndrome), metabolic diseases (e.g., autoimmune thyroiditis), gastrointestinal disorders (e.g., inflammatory bowel disease), vasculitides, and other autoimmune or inflammatory diseases [16].

### 2.5 *Measurement of anti-*Na*-APR-1 IgG antibodies*

Serum levels of *Na*-APR-1(M74)-specific IgG antibodies were determined using a qualified indirect ELISA utilizing homologous interpolation of the sample optical density (OD) values at 492 nm onto a Standard Calibration Curve (SCC). The SCC was derived from human standard reference sera (SRS) made by pooling serum samples obtained from participants with high levels of IgG to *Na*-APR-1 at study day 126 (i.e., 2 weeks following the third dose) as determined during an interim serological analysis of this study. Serial dilutions of the SRS were included in duplicate on each ELISA plate to produce a sigmoidal response curve, as required for the four-parameter logistic log model [17]. The SCCs were also used to calculate the IgG reactivity threshold used to determine IgG seroconversion to *Na*-APR-1(M74).

Arbitrary Units (AU) of anti-*Na*-APR-1(M74) IgG were derived as described previously [18], [19]. In brief, 1:1000 dilutions of test samples were added to 96-well microtiter plates (Nunc Maxisorb) in duplicate. Horseradish peroxidase conjugate secondary antibodies were added at 1:1,000 for IgG (Southern Biotech). All ELISA plates were developed in the dark with o-phenylenediamine dihydrochloride (OPD) (Sigma Aldrich) for 30 min at room temperature and read at 492 nm on a SpectraMax Plus 384 Microplate Reader (Molecular Devices). SOFTmax GXP PRO version 4 (Molecular Devices) was used to collect data. Average test sera ODs were then interpolated onto the SCC to derive the AU values of anti-*Na*-APR-1(M74) IgG. A Limit of Quantitation (LOQ) of 5.86 AU for the anti-*Na*-APR-1(M74) IgG assay was calculated from the SCCs per the method described in [19].

### 2.6 Statistical analysis

Percentages of study participants who experienced each adverse event were tabulated by dose and formulation [with or without GLA-AF] of *Na*-APR-1(M74). Fisher’s Exact Test was used to test for differences in the frequencies of adverse events between groups given the low numbers of most adverse events.

Non-parametric Mann-Whitney-Wilcoxon U tests were used to analyze differences in IgG antibody levels to *Na*-APR-1(M74) between dose groups by study day. If a statistically significant result (p < 0.05) was obtained, individual pair-wise tests were subsequently performed, with significance determined using the Holm-Bonferroni adjustment. Kruskal-Wallis tests were used to compare changes in antibody levels between participants on each study day. SAS software (version 9.3; SAS Institute) was used for all statistical analyses.

## 3 Results

### 3.1 Baseline Data and participant flow

**Fig. 1:**
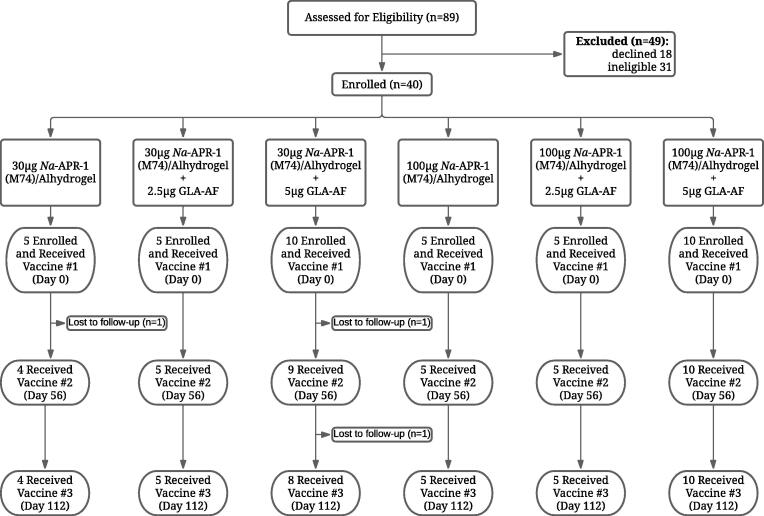
Study flow diagram.

Of 89 adults screened for enrollment in this Phase 1 trial of *Na*-APR-1(M74)/Alhydrogel, 18 decided against participation or were lost to follow-up before randomization, 31 were not eligible, and 40 (28 males and 12 females) qualified according to eligibility criteria and were randomized into the study (Fig. 1). Reasons for exclusion included: 1) being unavailable for all planned study visits (n = 3); 2) concomitant conditions such as severe asthma, uncontrolled hypertension, substance abuse, major psychiatric illness, and poor venous access (n = 12); and, 3) abnormal clinical laboratory tests (n = 18). The mean age of enrolled participants was 34.75 years (range, 20–50, SD = 9.55). The most prevalent race reflected in the total group was Black or African American (n = 25, 62.5 %), followed by White (n = 9, 22.5 %).

Of the 40 individuals who were included in the trial, 2 were lost to follow-up or withdrew for personal reasons after receiving the first vaccination, whereas 1 withdrew for personal reasons (moved from study area) after receipt of the first two vaccinations. No participants withdrew due to experiencing an adverse event. Five participants were lost to follow-up after receiving the third vaccination but before the final study visit at study day 470.

Baseline age and gender for enrolled study participants are included as Supplementary Information (**Supplementary** Table S1).

### 3.2 Safety

The *Na*-APR-1(M74)/Alhydrogel vaccine was tolerated well, regardless of whether it was co-administered with GLA-AF. None of the study participants withdrew or had their vaccinations discontinued because of significant reactogenicity, unsolicited adverse events, or abnormal clinical laboratory results. No vaccine-related serious adverse events (SAEs) occurred, and no vaccine recipient developed an adverse event of special interest. One SAE was recorded in a participant vaccinated with 30 µg *Na*-APR-1(M74)/Alhydrogel plus 2.5 µg GLA-AF. This volunteer was hospitalized for one night, approximately 1.5 months after receiving the second vaccination, for parenteral correction of hypocalcemia that was likely due to a previously undisclosed diagnosis of hypoparathyroidism. The low calcium level was discovered incidentally and was asymptomatic. This SAE was judged to be unrelated to vaccination.

The most frequent injection site reactions were mild tenderness and pain (Table 1), which were observed in up to 80% of participants per injection per vaccine group. However, 3 severe (grade 3) injection site reactions were reported: 2 occurrences of severe injection site tenderness, and 1 incident of severe injection site pain. For each of these subjects, the severe reactions were reported for only 1 of the 3 injections. Each of these 3 subjects were in a different vaccine group: 1 experienced the severe reaction after receiving the third administration of 30 µg *Na*-APR-1(M74)/Alhydrogel plus 2.5 µg GLA-AF, 1 after receiving the second administration of 100 µg *Na*-APR-1(M74)/Alhydrogel plus 2.5 µg GLA-AF, and 1 after receiving the second administration of 100 µg *Na*-APR-1(M74)/Alhydrogel plus 5.0 µg GLA-AF. A 2x3 Fisher’s Exact Test revealed no statistically significant relation between injection site pain (of any severity) and GLA-AF status (p = 0.727) or between injection site tenderness and GLA-AF status (p = 0.424). Overall, injection site reactions resolved in a median of 1 day (range, 0 to 10).

**Table 1:** **SolicitedInjectionSite andSystemicAdverseEvents afterVaccination with *Na*-APR-1(M74)/Alhydrogel (with or without GLA-AF) inHookworm-naïveAdults.** Data are number (%) of participants experiencing an event after any vaccination. Groups receiving either of the 2 doses of GLA-AF were combined. Participants with more than one occurrence of the same adverse event are recorded only once, at the maximum severity experienced.

	**30 µg *Na*-APR-1(M74)/Alhydrogel** (n = 5)			**30 µg *Na*-APR-1(M74)/Alhydrogel + GLA-AF** (n = 15)			**100 µg *Na*-APR-1(M74)/Alhydrogel** (n = 5)			**100 µg *Na*-APR-1(M74)/Alhydrogel + GLA-AF** (n = 15)		
	Mild	Moderate	Severe	Mild	Moderate	Severe	Mild	Moderate	Severe	Mild	Moderate	Severe
	N (%)	N (%)	N (%)	N (%)	N (%)	N (%)	N (%)	N (%)	N (%)	N (%)	N (%)	N (%)
**Local**												
Pain	4 (80)	0	0	12 (80)	1 (6.7)	0	3 (60)	1 (20)	0	8 (53)	6 (40)	1 (6.7)
Tenderness	2 (40)	0	0	7 (47)	2 (13)	1 (6.7)	2 (40)	0	0	6 (40)	8 (53)	1 (6.7)
Swelling	0	0	0	0	0	0	0	0	0	3 (20)	0	0
Erythema	0	0	0	1 (6.7)	0	0	0	0	0	1 (6.7)	0	0
**Systemic**												
Headache	2 (40)	1 (20)	0	3 (20)	1 (6.7)	2 (13)	2 (40)	0	0	6 (40)	0	0
Nausea	1 (20)	0	0	3 (20)	1 (6.7)	1 (6.7)	1 (20)	0	0	2 (13)	0	0
Vomiting	0	0	0	3 (20)	1 (6.7)	1 (6.7)	0	0	0	1 (6.7)	0	0
Myalgia	1 (20)	0	0	0	0	0	1 (20)	0	0	2 (13)	3 (20)	0
Arthralgia	1 (20)	0	0	1 (20)	0	0	2 (40)	0	0	1 (6.7)	1 (6.7)	0
Fever	0	0	0	1 (6.7)	1 (6.7)	0	0	0	0	0	0	0
Urticaria	0	0	0	0	0	0	0	0	0	0	0	0

When comparing injection site reactions, moderate and severe reactions were more common in participants who received *Na*-APR-1(M74)/Alhydrogel co-administered with GLA-AF: whereas only 1 participant (10%) receiving *Na*-APR-1(M74)/Alhydrogel *without* GLA-AF reported a moderate injection site reaction (pain), this increased to 40% for those to whom the vaccine was co-administered with 5 µg GLA-AF, although this finding was not statistically significant, possibly due to the study’s small sample size.

The most commonly reported solicited systemic events occurring within 14 days of vaccination were mild headache and nausea (Table 1). Mild systemic reactions (grade 1) were observed in up to 60% of participants per injection per vaccine group. Stratified by GLA-AF status, regardless of *Na*-APR-1(M74) dose, maximum incidence rates of mild systemic reactions were 33% per injection, or 50 % for all vaccinations (Table 2). Six severe (grade 3) systemic reactions occurred in 2 participants: 2 cases of headache, 2 of nausea, and 2 of vomiting. All 6 severe systemic reactions were deemed possibly related to study vaccine. Five of these events were reported in one participant vaccinated with 30 µg *Na*-APR-1(M74)/Alhydrogel plus 5 µg GLA-AF (severe nausea and vomiting 1 day after the second vaccination and severe headache, nausea and vomiting starting 5 days after the third vaccination) and 1 event (headache) in a participant after receiving the first dose of 30 µg *Na*-APR-1(M74)/Alhydrogel plus 2.5 µg GLA-AF. No severe systemic reactions were reported for participants vaccinated without GLA-AF or with 100 µg *Na*-APR-1(M74)/Alhydrogel (with or without GLA-AF).

**Table 2:** **Incidence of Solicited SystemicAdverse Events by GLA-AF Status for All Vaccinations.** Data are number (%) of participants experiencing an event after any vaccination. Results for the different doses of *Na*-APR-1(M74) for the same dose of GLA-AF are combined. Participants with more than one occurrence of the same adverse event are recorded only once, at the maximum severity experienced.

	***Na*-APR-1(M74)/Alhydrogel without GLA-AF** (n = 10)			***Na*-APR-1(M74)/Alhydrogel +** **2.5 µg GLA-AF** (n = 10)			***Na*-APR-1(M74)/Alhydrogel +** **5 µg GLA-AF** (n = 20)		
	Mild	Moderate	Severe	Mild	Moderate	Severe	Mild	Moderate	Severe
	N (%)	N (%)	N (%)	N (%)	N (%)	N (%)	N (%)	N (%)	N (%)
Arthralgia	3 (30.0 %)	0	0	0	2 (20.0 %)	0	2 (10.0 %)	0	0
Headache	4 (40.0 %)	1 (10.0 %)	0	5 (50.0 %)	0	1 (10.0 %)	4 (20.0 %)	1 (5.0 %)	1 (5.0 %)
Myalgia	2 (20.0 %)	0	0	2 (20.0 %)	2 (20.0 %)	0	0	1 (5.0 %)	0
Nausea	2 (20.0 %)	0	0	1 (10.0 %)	1 (10.0 %)	0	4 (20.0 %)	0	1 (5.0 %)
Vomiting	0	0	0	0	1 (10.0 %)	0	4 (20.0 %)	0	1 (5.0 %)
Fever	0	0	0	0	1 (10.0 %)	0	1 (5.0 %)	0	0

The incidence of severe solicited systemic reactions, particularly headache and nausea, increased slightly when GLA-AF was co-administered with *Na*-APR-1(M74)/Alhydrogel, as shown in Table 2. However, significant differences in the frequency of these events were not observed between those vaccinated with *Na*-APR-1(M74)/Alhydrogel plus GLA-AF and those vaccinated with *Na*-APR-1(M74)/Alhydrogel alone. Statistical comparisons between the frequencies of other solicited systemic adverse events could not be made due to their low numbers.

Decreases in hemoglobin from baseline (i.e., the day of the first vaccination) were the most frequently observed clinical laboratory abnormalities (Table 3). Thirteen of 40 (32.5%) participants had at least 1 drop in hemoglobin from baseline during 16 months of follow-up: of these, 12 were mild (a decrease of 1.0 to 1.5 g/dl), whereas 1 was moderate (a reduction of 1.9 g/dl between study days 0 and 126, although all values remained above the lower reference limit for the clinical site) in a participant who received 3 doses of 100 µg *Na*-APR-1(M74)/Alhydrogel plus 5 µg GLA-AF. Of these, only 1, a male participant in the 100 µg *Na*-APR-1(M74)/Alhydrogel plus 2.5 µg GLA-AF group, had a hemoglobin concentration (12.3 g/dl) that was under the lower reference limit (12.5 g/dl) on study day 126, which was defined by the protocol as a mild (Grade 1) adverse event; however, this event was assessed by the investigators as unlikely related to vaccination. No significant differences in frequencies of reductions in hemoglobin concentration were identified between doses of *Na*-APR-1(M74), or co-administration status with GLA-AF. The most common other clinical laboratory abnormalities observed were mild (grade 1) decreases in absolute neutrophil count in 5 participants, and mild increases in alanine aminotransferase concentration, also in 5 participants. The observed clinical laboratory test abnormalities were all asymptomatic and transient, and there were no differences between dose concentrations of *Na*-APR-1(M74) or co-administration status with GLA-AF.

**Table 3:** **ClinicalLaboratoryAdverseEvents afterVaccination with *Na*-APR-1(M74)/Alhydrogel (with or without GLA-AF) inHookworm-naïveAdults.** Data are number (%) of participants experiencing an event after any vaccination. Results for the different doses of GLA-AF for the same dose of *Na*-APR-1(M74) are combined. Participants with more than one occurrence of the same adverse event are recorded only once, at the maximum severity experienced.

	**30 µg *Na*-APR-1(M74)/Alhydrogel**® (n = 5)		**30 µg *Na*-APR-1(M74)/Alhydrogel + GLA-AF** (n = 15)		**100 µg *Na*-APR-1(M74)/Alhydrogel** (n = 5)		**100 µg *Na*-APR-1(M74)/Alhydrogel + GLA-AF** (n = 15)	
	Mild	Moderate/ Severe	Mild	Moderate/ Severe	Mild	Moderate/ Severe	Mild	Moderate
	N (%)	N (%)	N (%)	N (%)	N (%)	N (%)	N (%)	N (%)
Low Hemoglobin Concentration	0	0	0	0	0	0	1 (6.7)	0
Reduction in Hemoglobin from Baseline*	3 (60)	0	2 (13)	0	1 (20)	0	6 (40)	1 (6.7)
Increased WBC	0	0	0	0	0	0	1 (6.7)	0
Decreased WBC	1 (20)	0	2 (13)	0	1 (20)	0	2 (13)	0
Decreased ANC	1 (20)	0	2 (13)	0	0	0	1 (6.7)	0
Decreased Platelets	0	0	1 (6.7)	0	0	0	0	1 (6.7)
Increased ALT	0	0	4 (27)	0	0	0	1 (6.7)	0
Increased Creatinine	0	0	1 (6.7)	0	0	0	1 (6.7)	0

WBC, white blood cell count; ANC, absolute neutrophil count; ALT, alanine aminotransferase.

* From Study Day 0.

A total of 55 (49 mild; 2 moderate; 4 severe) unsolicited adverse events were reported in 28 (70%) participants that were judged to be definitely, probably, or possibly related to study product. Unsolicited adverse events assessed as being related to study vaccine and that were considered severe (grade 3) were reported for 2 subjects: severe fatigue and decreased appetite were reported in 1 participant starting 3 days after the first vaccination with 30 µg *Na*-APR-1(M74)/Alhydrogel plus 2.5 µg GLA-AF and coincided with symptoms consistent with an upper respiratory tract infection (cough and sore throat), whereas severe abdominal pain and chills (associated with nausea and vomiting) were reported in 1 participant starting 6 days after the third vaccination with 30 µg *Na*-APR-1(M74)/Alhydrogel plus 5 µg GLA-AF; all of these events lasted 2 days or less, and were self-limited, resolving completely without complications or the need for treatment. No differences were found in the incidence of vaccine-related unsolicited adverse events between vaccine groups or by GLA-AF status, although no formal statistics were performed given the small number of events.

### 3.3 *IgG antibody responses to* Na*-APR-1(M74)*

**Fig. 2:**
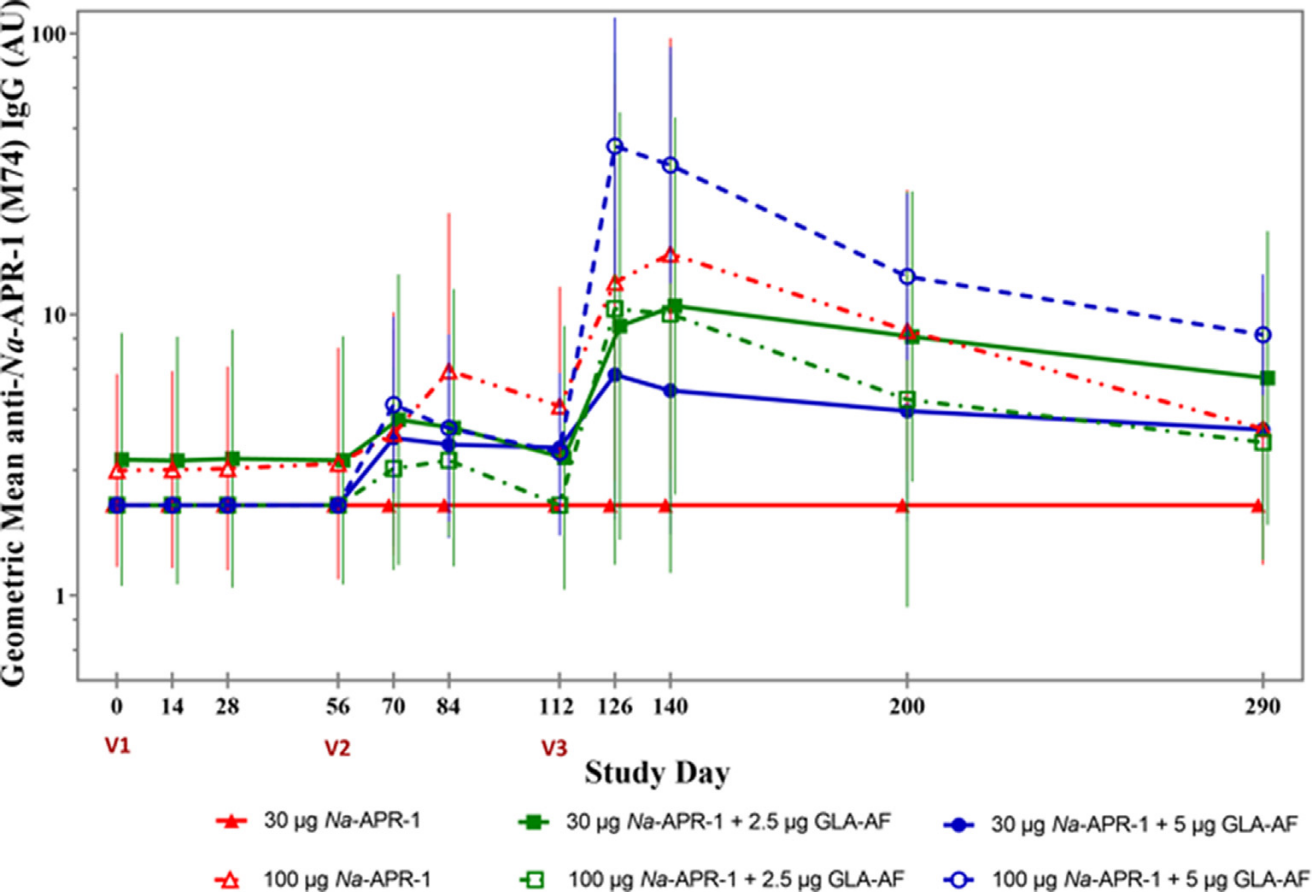
**GeometricMeanLevels of IgG againstRecombinant *Na*-APR-1(M74) asMeasured by ELISA.** Bars represent 95% confidence intervals. V1 = first vaccination; V2 = second vaccination; V3 = third vaccination; AU = arbitrary units.

Anti-*Na*-APR-1(M74)-specific IgG antibody levels were below the LOQ of 5.86 AUs in all but 2 participants at baseline (1 in the 30 µg *Na*-APR-1(M74)/Alhydrogel + 2.5 µg GLA-AF group [13.6 AU] and 1 in the 100 µg *Na*-APR-1(M74)/Alhydrogel group [8.7 AU]) prior to administration of study vaccine (Table 4). Overall, levels of anti-*Na*-APR-1 IgG increased with successive administrations of the vaccine, peaking on study day 126 or 14 days after the third and final vaccination (Fig. 2). Geometric mean levels of *Na*-APR-1-specific IgG dropped thereafter, although they remained higher than baseline levels for the remainder of the study (12 months). Administration of *Na*-APR-1(M74)/Alhydrogel either with or without GLA-AF resulted in production of antigen-specific IgG antibodies in a dose-dependent manner; furthermore, increasing antibody levels were measured after successive vaccinations (Fig. 2). No detectable antigen-specific IgG antibodies were measured at any study visit in the 30 µg *Na*-APR-1(M74)/Alhydrogel group, indicating a lack of immunogenicity. However, IgG antibodies were detected post-vaccination in all other groups, including those administered 30 µg *Na*-APR-1(M74)/Alhydrogel with GLA-AF.

**Table 4:** **ELISA Mean anti-*Na*-APR-1(M74) IgG Levels and Percent Responders by Vaccine Group, Per Protocol Population.** Participants with values above the limit of quantitation (5.86 AU) were considered seropositive.

**Study Day**	**30 µg *Na*-APR-1(M74)/Alhydrogel**									**100 µg *Na*-APR-1(M74)/Alhydrogel**								
	**0 µg GLA-AF**			**2.5 µg GLA-AF**			**5 µg GLA-AF**			**0 µg GLA-AF**			**2.5 µg GLA-AF**			**5 µg GLA-AF**		
	Mean (SD)	Range	Responders (%)	Mean (SD)	Range	Responders (%)	Mean (SD)	Range	Responders (%)	Mean (SD)	Range	Responders (%)	Mean (SD)	Range	Responders (%)	Mean (SD)	Range	Responders (%)
Day 0	2.1 (0)	2.1–2.1	0/4 (0)	4.4 (5.13)	2.1–13.6	1/5 (20)	2.1 (0)	2.1–2.1	0/8 (0)	3.4 (2.97)	2.1–8.7	1/5 (20)	2.1 (0)	2.1–2.1	0/5 (0)	2.1 (0)	2.1–2.1	0/10 (0)
Day 14	2.1 (0)	2.1–2.1	0/4 (0)	4.3 (4.91)	2.1–13.1	1/5 (20)	2.1 (0)	2.1–2.1	0/8 (0)	3.5 (3.08)	2.1–9	1/5 (20)	2.1 (0)	2.1–2.1	0/5 (0)	2.1 (0)	2.1–2.1	0/10 (0)
Day 28	2.1 (0)	2.1–2.1	0/4 (0)	4.5 (5.36)	2.1–14.1	1/5 (20)	2.1 (0)	2.1–2.1	0/8 (0)	3.6 (3.29)	2.1–9.5	1/5 (20)	2.1 (0)	2.1–2.1	0/5 (0)	2.1 (0)	2.1–2.1	0/10 (0)
Day 56	2.1 (0)	2.1–2.1	0/4 (0)	4.3 (4.93)	2.1–13.1	1/5 (20)	2.1 (0)	2.1–2.1	0/8 (0)	4 (4.24)	2.1–11.6	1/5 (20)	2.1 (0)	2.1–2.1	0/5 (0)	2.1 (0)	2.1–2.1	0/10 (0)
Day 70	2.1 (0)	2.1–2.1	0/4 (0)	6.1 (5.47)	2.1–12.1	2/5 (40	9.6 (17.43)	2.1–51.8	2/8 (25)	4.9 (3.84)	2.1–9.4	2/5 (40 %)	3.6 (3.27)	2.1–9.4	1/5 (20)	7.9 (9)	2.1–25.8	4/10 (40)
Day 84	2.1 (0)	2.1–2.1	0/4 (0)	5.7 (5.84)	2.1–15.5	2/5 (40)	6.7 (9.11)	2.1–26.5	2/8 (25)	9.1 (7.41)	2.1–19.4	3/5 (60 %)	3.4 (1.73)	2.1–5.3	0/5 (0)	7.4 (10.05)	2.1–29.4	3/10 (30)
Day 112	2.1 (0)	2.1–2.1	0/4 (0)	4.6 (5.64)	2.1–14.7	1/5 (20)	5.9 (7.13)	2.1–18	2/8 (25)	5.9 (4.01)	2.1–10.7	2/5 (40 %)	2.1 (0)	2.1–2.1	0/5 (0)	5.3 (6.88)	2.1–20.1	2/10 (20)
Day 126	2.1 (0)	2.1–2.1	0/4 (0)	17.3 (17.54)	2.1–42.9	3/5 (60)	18.5 (35.79)	2.1–105.8	4/8 (50)	24.7 (32)	2.1–81.1	4/5 (80)	28.5 (39.92)	2.1–96.2	3/5 (60 %)	117.2 (205.11)	7.7–660.5	10/10 (100)
Day 140	2.1 (0)	2.1–2.1	0/4 (0)	17.9 (17.07)	2.1–42.6	3/5 (60)	14.7 (25.11)	2.1–74	3/8 (37.5)	33.5 (44.64)	2.1–112.1	4/5 (80)	30.5 (48.49)	2.1–115.7	3/5 (60 %)	84.3 (137.93)	5.4–444.2	9/10 (90)
Day 200	2.1 (0)	2.1–2.1	0/4 (0)	11.4 (8.97)	2.1–23.5	4/5 (80)	7.7 (8.33)	2.1–23.7	3/8 (37.5)	11.6 (8.19)	2.1–22.7	4/5 (80)	7.6 (7.91)	2.1–18.9	2/4 (50 %)	17.8 (11.32)	2.1–34.8	8/9 (88.9)
Day 290	2.1 (0)	2.1–2.1	0/4 (0)	8.2 (5.96)	2.1–15.5	3/5 (60)	6 (6.93)	2.1–21.7	3/8 (37.5)	5.6 (5.5)	2.1–14.7	2/5 (40)	4 (2.31)	2.1–6.7	1/4 (25)	10.2 (5.76)	2.1–19.5	7/10 (70)

No antibody responses were detected in any vaccine dose group after the first administration of *Na*-APR-1/(M74)/Alhydrogel with or without GLA. The highest antibody responses were seen after the second vaccination in subjects who received 30 µg *Na*-APR-1(M74)/Alhydrogel co-administered with 5.0 µg GLA-AF. Only 25% of these subjects (2 out of 8), however, were seropositive at this time point. The highest antibody response after the third administration of the vaccine was seen in those vaccinated with 100 µg *Na*-APR-1(M74)/Alhydrogel co-administered with 5.0 µg GLA-AF. This result is in line with the highest percentage of subjects considered seropositive after the third administration of the vaccine: 100% of the subjects vaccinated with 100 µg *Na*-APR-1(M74)/Alhydrogel co-administered with 5.0 µg GLA-AF were seropositive 14 days post-administration. At 6 months following the third administration of the vaccine, 70% of these subjects were still seropositive, versus 0–60% of those in the other vaccine groups. Statistically significant differences between the 30 µg *Na*-APR-1(M74)/Alhydrogel versus the 100 µg *Na*-APR-1(M74)/Alhydrogel groups were seen on study days 126 and 140 (Mann-Whitney test, p = 0.006 and p = 0.008, respectively), regardless of GLA-AF status. A pairwise test (Mann-Whitney with Holm-Bonferroni correction) showed a significant difference between the 30 µg *Na*-APR-1(M74)/Alhydrogel versus the 100 µg *Na*-APR-1(M74)/Alhydrogel + 5 µg GLA-AF group at days 126 and 140, but not at the other study days or for other pairs. 100 µg *Na*-APR-1(M74)/Alhydrogel co-administered with 5.0 µg GLA-AF was the dose and formulation that generated the highest level of anti-*Na*-APR-1(M74) antibodies after vaccination.

## 4 Discussion

Herein we report the results of the first Phase 1 clinical trial of the novel *Na*-APR-1 (M74)/Alhydrogel vaccine targeting the adult blood-feeding stage of *N. americanus* hookworms. In this trial, *Na*-APR-1(M74)/Alhydrogel was safe and well tolerated in healthy adults who are resident in the US and as such, most likely naïve to hookworm infection. Mild injection site pain and tenderness, headache, and nausea were the most common solicited symptoms. Solicited injection site and systemic symptoms occurred at similar rates between groups, although a possible trend was observed for increased severity of solicited symptoms among those who received of *Na*-APR-1(M74)/Alhydrogel plus GLA-AF compared to those who received *Na*-APR-1(M74)/Alhydrogel without GLA-AF. While the proportion of participants experiencing moderate or severe injection site pain or tenderness, headache, and nausea in those receiving *Na*-APR-1(M74)/Alhydrogel with GLA-AF did not appear to differ from the *Na*-APR-1(M74)/Alhydrogel without GLA-AF group, the small numbers of participants in each group precluded a statistically robust analysis of trends in the occurrence of these events. Clinical laboratory adverse events deemed to be vaccine-related were few in number; furthermore, these abnormalities were asymptomatic, mild, and self-limited.

The *Na*-APR-1(M74)/Alhydrogel vaccine induced antigen-specific IgG responses in a dose-dependent manner, and increasingly higher peaks of antibody were observed with each successive vaccination. Co-administering GLA-AF with *Na*-APR-1(M74)/Alhydrogel augmented the antigen-specific IgG responses at each of the two vaccine antigen concentrations that were tested in this trial, although this effect was not statistically significant, possibly due to the small group sizes. Although conducted in a different population, the IgG responses observed in this hookworm-unexposed individuals were similar to those seen in a Phase 1 trial in Gabonese adults residing in a hookworm-endemic area in which *Na*-APR-1(M74)/Alhydrogel was co-administered with the *Na*-GST-1/Alhydrogel candidate vaccine [17].

Unexpectedly, two participants had detectable albeit low levels of IgG against *Na*-APR-1(M74) at baseline. Additional investigations would be required to investigate if these individuals had antibodies that specifically bind to recombinant *Na*-APR-1(M74), or rather, are cross-reactive with similar proteins to which they had previously been exposed. Not surprisingly, the baseline prevalence of seropositivity to *Na*-APR-1(M74) was considerably lower than that seen in the trial conducted in a hookworm-endemic region of Gabon, in which 54 % of participants had detectable levels of IgG to the recombinant protein by ELISA [17].

In the digestive tract of adult *N. americanus* hookworms, an ordered sequence of enzymes is used by the parasite to process ingested host hemoglobin into the smaller peptides that are essential nutrients for this helminth, an obligate blood feeder. *Na*-APR-1, a cathepsin D aspartic protease, is the first enzyme in this hemoglobin digestion cascade. This enzyme is a promising hookworm vaccine candidate, as administration of the canine version of *Na*-APR-1 (*Ac*-APR-1) induced antibodies in dogs that neutralized native *Ac*-APR-1 activity *in vivo* and also bound to the digestive tract of worms used to produce controlled challenge infections [13], [14]. In this experiment, vaccination with recombinant *Ac*-APR-1 resulting in significantly reduced parasite burdens and blood loss compared to unvaccinated dogs.

However, manufacture of recombinant *Na*-APR-1 has been challenging. First, since native *Na*-APR-1 can enzymatically cleave human hemoglobin, for safety reasons it was expressed as a catalytically inactivated mutant protein in which 2 aspartic acid residues were substituted for alanines to produce *Na*-APR-1(M74). Although refolded *Na*-APR-1 (M74) can be expressed in *Escherichia coli*, significant protein aggregation results in poor yield of a monomeric product that would be suitable for testing in humans [13]. As an alternative strategy, the recombinant *Na-*APR-1(M74) that was tested in the clinical trial described herein was derived by infiltration of *Agrobacterium tumefaciens* strain GV3101, genetically altered to express *Na-*APR-1(M74) in *Nicotiana benthamiana* tobacco plants [15]*.* A potential limitation of this approach, however, may be the barrier to scale-up manufacture, since this has never been done using this technology, to date.

*Na*-APR-1(M74) is the third human hookworm vaccine candidate to be tested in a clinical trial. The first recombinant hookworm vaccine administered to humans contained recombinant *Na*-ASP-2 (Ancylostoma Secreted Protein-2 of *N. americanus*) as the active ingredient. As reported previously, when administered to Brazilian adults who had recently been treated for hookworm infection, generalized urticaria was induced in several participants immediately following the first vaccination due to presence of IgE to the vaccine antigen likely induced by prior hookworm infection [18]. Given this experience, further clinical development of hookworm vaccine antigens has been narrowed to focus on those that are not associated with significant levels of IgE antibodies from natural infection.

Population-based serosurveys in hookworm-endemic regions of Brazil demonstrated that a significant proportion of individuals, from a wide range of ages, have detectable levels of IgE to *Na*-ASP-2 and several additional antigens expressed primarily during the larval stage of infection [18]. Due to this precedent, prior to initiating clinical development of the *Na*-APR-1 antigen, field studies were conducted to evaluate whether IgE antibodies were induced to this vaccine candidate by natural infection with *N. americanus*. In one representative study, sera from 648 hookworm-infected individuals aged 6 years and older from hookworm-endemic areas of Minas Gerais state, Brazil, were tested for antibodies to *Na*-APR-1(M74) using an indirect ELISA, with only 0.6% having detectable antigen-specific IgE antibodies [14]. Therefore, clinical development of this product is unlikely to result in the type of immediate-type hypersensitivity reactions that were observed when *Na*-ASP-2 was tested in individuals who had previously been infected with hookworm. Indeed, no allergic reactions were observed in the study reported herein.

Children living in hookworm-endemic regions are often infected with hookworm at a young age and may suffer from chronic infection throughout childhood and into adulthood due to the years-long survival of worms in the intestinal tract and the lack of protective immunity against re-infection [20], [21], [22], [23]. Although the nature of the protective immune response that will be required for an effective hookworm vaccine is unknown, it will certainly differ in character from that associated with natural hookworm infection since this is not protective. Therefore, the goal of vaccination with *Na*-APR-1(M74)/Alhydrogel is not to replicate the immune response observed in those infected with *N. americanus*; instead, it should elicit IgG antibodies that are ingested during a blood feed by adult hookworms in the host intestine. These antibodies will interfere with the parasite’s ability to cleave heme into digestible peptides, and thereby starve the worm. A parallel mechanism of action is proposed for the recombinant *Na*-GST-1 hookworm vaccine antigen [24], which interferes with the final steps in the blood digestion cascade of *N. americanus*. Vaccine-induced antibodies against *Na*-GST-1 are also ingested by blood-feeding hookworms and impede the parasite’s ability to detoxify free heme by native *Na*-GST-1, resulting in the accumulation of heme to toxic levels. Combining these two vaccine antigens – *Na*-APR-1(M74) and *Na*-GST-1 – would therefore interfere with the entire blood digestion pathway. It is hoped that this two-pronged attack will have an additive if not synergistic effect on vaccine efficacy and serves as the rationale for developing the Human Hookworm Vaccine as a combination of these two candidates.

Given the putative mechanism of action outlined above, the goal of formulating the *Na*-APR-1(M74) antigen has been to optimize levels of vaccine-induced IgG antibodies that would neutralize the action of native *Na*-APR-1 and interfere with the critical blood-feeding pathway of *N. americanus* hookworms. Accordingly, the *Na*-APR-1/Alhydrogel vaccine was evaluated when co-administered with an aqueous formulation of the GLA immunostimulant in the clinical trial reported in this manuscript. The active component of GLA-AF is synthetic monophosphoryl lipid A (MPL), a derivative of *Salmonella minnesota* endotoxin, a TLR4 agonist [25]. In the trial reported herein, mixing GLA-AF with *Na*-APR-1(M74)/Alhydrogel prior to injection resulted in higher antigen-specific IgG responses at both the 30 µg and 100 µg dose levels of *Na*-APR-1(M74), although the differences were statistically insignificant, in all probability because of the small number of participants in each vaccine group. This contrasts with the clinical experience with the *Na*-GST-1/Alhydrogel hookworm vaccine candidate, for which the addition of GLA-AF as an adjuvant did not result in any improvement in antigen-specific IgG responses when tested in either hookworm-naïve or hookworm-exposed adults [26]. Given that the eventual goal for the Human Hookworm Vaccine as stated above is to co-formulate the *Na*-GST-1 and *Na*-APR-1(M74) antigens as a single product, the differential effect of this adjuvant on antigen immunogenicity raises questions of its utility in future formulations.

In conclusion, in this first-in-humans Phase 1 vaccine trial, the *Na*-APR-1(M74)/Alhydrogel vaccine was safe, well-tolerated and immunogenic in adults who were hookworm-naïve. The vaccine elicited antigen-specific IgG antibodies in a dose-dependent manner. Co-administration with the GLA-AF immunostimulant augmented the humoral immune response to the *Na*-APR-1 antigen, although the level of antibodies that will be required for protection remains to be established in larger Phase 2 and 3 clinical field trials or controlled human hookworm infection (CHHI) studies. Given the results presented herein, further studies of this candidate vaccine have been undertaken in adults [17] and children living in endemic areas of sub-Saharan Africa, and preparations are underway to conduct proof-of-concept CHHI studies in Brazil to test the efficacy of this vaccine candidate in combination with *Na*-GST-1.

## Declaration of Competing Interest

The authors declare the following financial interests/personal relationships which may be considered as potential competing interests: David Diemert has patent Multivalent Antihelminthic Vaccine issued to The Albert B. Sabin Vaccine Institute, The George Washington University, The Council of the Queensland Institute of Medical Research. Peter J. Hotez has patent Multivalent Antihelminthic Vaccine issued to The Albert B. Sabin Vaccine Institute, The George Washington University, The Council of the Queensland Institute of Medical Research. Jeffrey Bethony has patent Multivalent Antihelminthic Vaccine issued to The Albert B. Sabin Vaccine Institute, The George Washington University, The Council of the Queensland Institute of Medical Research. Maria Elena Bottazzi has patent Multivalent Antihelminthic Vaccine issued to The Albert B. Sabin Vaccine Institute, The George Washington University, The Council of the Queensland Institute of Medical Research. Peter J. Hotez has patent Human Hookworm Vaccine issued to The George Washington University. Maria Elena Bottazzi has patent Human Hookworm Vaccine issued to The George Washington University. Jeffrey Bethony has patent Human Hookworm Vaccine pending to The George Washington University.
