# Supplementary material for: Safety and immunogenicity of the Na-APR-1 hookworm vaccine in infection-naïve adults
Source: Vaccine. 2022 Oct 6;40(42):6084–92. doi: 10.1016/j.vaccine.2022.09.017 (PMC9549940; doi:10.1016/j.vaccine.2022.09.017)
Supplement: Supplementary data 1 [file mmc1.docx]

**Supplementary Material**

**Table S1. Summary of Demographic Characteristics by Vaccine Allocation Group**

Summaries are count (percent) for categorical variables and mean (SD) for numeric variables.

|  |  | **30 µg *Na*-APR-1(M74)/Alhydrogel** | | | **100 µg *Na*-APR-1(M74)/Alhydrogel** | | | **All Participants** |
| --- | --- | --- | --- | --- | --- | --- | --- | --- |
| **Variable** | **Characteristic** | 0 µg GLA-AF  (N=5) | 2.5 µg GLA-AF  (N=5) | 5 µg GLA-AF  (N=10) | 0 µg GLA-AF  (N=5) | 2.5 µg GLA-AF  (N=5) | 5 µg GLA-AF  (N=10) | (N=40) |
| Sex | Female | 0 (0) | 2 (40) | 4 (40) | 1 (20) | 0 (0) | 5 (50) | 12 (30) |
|  | Male | 5 (100) | 3 (60) | 6 (60) | 4 (80) | 5 (100) | 5 (50) | 28 (70) |
| Ethnicity | Not Hispanic or Latino | 4 (80) | 5 (100) | 10 (100) | 5 (100) | 4 (80) | 10 (100) | 38 (95) |
|  | Hispanic or Latino | 1 (20) | 0 (0) | 0 (0) | 0 (0) | 1 (20) | 0 (0) | 2 (5) |
| Race | American Indian or Alaska Native | 0 (0) | 0 (0) | 0 (0) | 0 (0) | 0 (0) | 0 (0) | 0 (0) |
|  | Asian | 0 (0) | 0 (0) | 1 (10) | 0 (0) | 0 (0) | 0 (0) | 1 (2.5) |
|  | Native Hawaiian or Other Pacific Islander | 0 (0) | 0 (0) | 1 (10) | 0 (0) | 0 (0) | 0 (0) | 1 (2.5) |
|  | Black or African American | 4 (80) | 3 (60) | 6 (60) | 4 (80) | 4 (80) | 4 (40) | 25 (62.5) |
|  | White | 1 (20) | 2 (40) | 1 (10) | 0 (0) | 0 (0) | 5 (50) | 9 (22.5) |
|  | Multi-Racial | 0 (0) | 0 (0) | 1 (10) | 1 (20) | 1 (20) | 1 (10) | 4 (10) |
| Age (years) | Mean (standard deviation) | 42.5 (3.2) | 35.2 (11.3) | 35.7 (11.4) | 35.6 (8.5) | 34.4 (12.6) | 29.3 (5.5) | 34.8 (9.6) |
